# Positive Imagery Cognitive Bias Modification in Treatment-Seeking Patients with Major Depression in Iran: A Pilot Study

**DOI:** 10.1007/s10608-014-9598-8

**Published:** 2014-02-07

**Authors:** Hajar Torkan, Simon E. Blackwell, Emily A. Holmes, Mehrdad Kalantari, Hamid Taher Neshat-Doost, Mohsen Maroufi, Hooshang Talebi

**Affiliations:** 1 Isfahan Science and Research Branch, Islamic Azad University, Isfahan, Iran; 2 MRC Cognition and Brain Sciences Unit, Cambridge, UK; 3 Department of Psychology, University of Isfahan, Isfahan, Iran; 4 Isfahan University of Medical Sciences, Isfahan, Iran; 5 Department of Statistics, University of Isfahan, Isfahan, Iran

**Keywords:** Mental imagery, Cognitive bias modification, Depression, Computerized interventions, Interpretive bias

## Abstract

Cognitive bias modification paradigms training positive mental imagery and interpretation (imagery CBM-I) hold promise for treatment innovation in depression. However, depression is a global health problem and interventions need to translate across settings and cultures. The current pilot study investigated the impact of 1 week of daily imagery CBM-I in treatment-seeking individuals with major depression in outpatient psychiatry clinics in Iran. Further, it tested the importance of instructions to *imagine* the positive training materials. Finally, we examined the effects of this training on imagery vividness. Thirty-nine participants were randomly allocated to imagery CBM-I, a non-imagery control program, or a no treatment control group. Imagery CBM-I led to greater improvements in depressive symptoms, interpretive bias, and imagery vividness than either control condition at post-treatment (*n* = 13 per group), and improvements were maintained at 2-week follow-up (*n* = 8 per group). This pilot study provides first preliminary evidence that imagery CBM-I could provide positive clinical outcomes in an Iranian psychiatric setting, and further that the imagery component of the training may play a crucial role.

## Introduction

Depression is a global health problem (World Health Organization 2008), and thus new treatments need to translate worldwide. A major barrier to treating depression is limited access to psychological therapies, and the development of novel psychological interventions is a crucial area for research (Wittchen 2012). Cognitive science offers a means for the development of such new interventions via the identification of key processes involved in the maintenance of disorders and the means to modify these. A body of research has demonstrated that depression is characterized by negative biases in many aspects of processing including memory, attention, and interpretation (e.g. Mathews and MacLeod 2005; Gotlib and Joormann 2010; Everaert et al. 2012). Paradigms that aim to directly modify such biases, referred to as “cognitive bias modification” (CBM; MacLeod et al. 2009; Hertel and Mathews 2011) hold promise as novel accessible interventions for depression. However, the investigation of CBM for depression is in its infancy, with research tending to focus on anxiety (see e.g. MacLeod and Mathews 2012). CBM research in depression has also been largely restricted to European and English-speaking countries. If CBM paradigms are to help tackle a global problem such as depression then they need have global applicability. Thus a key question at this early stage of clinical research is whether a CBM paradigm developed in one setting can be successfully translated to and applied in a different country and culture.

A CBM paradigm that has shown some early promise as a potential intervention in depression is an adapted version of the interpretation training paradigm originally developed by Mathews and Mackintosh (2000). Participants are repeatedly presented with scenarios that start ambiguous, but are then resolved positively, with the aim of training a bias to automatically expect positive resolutions for novel ambiguous situations. Depression is characterised by the tendency to interpret ambiguous information negatively, a negative interpretation bias (Butler and Mathews 1983), and thus such training may be beneficial. In adapting the interpretation CBM (CBM-I) to depression there has been a particular focus on the use of mental imagery (Holmes et al. 2009a). Participants are required to imagine themselves in the scenarios presented, “as if actively involved, seeing them through your own eyes”, and early experimental studies have demonstrated the crucial role of this use of imagery in the effects of the CBM-I paradigm over a single session of training in non-clinical samples (Holmes et al. 2006; Holmes et al. 2008a, 2009b). However, when applied to depression the requirement to *imagine* the positive resolutions of the training scenarios may have even greater importance in reducing the clinical symptoms of the disorder.

Depression is characterised by a deficit in positive future imagery (Holmes et al. 2008b; Morina et al. 2011), such that people with depression may struggle to imagine anything other than negative possibilities in their future. Repeated practice in generating positive mental imagery may therefore be particularly helpful. In addition, depression is characterised by a bias for a verbal, ruminative style of processing (Koster et al. 2011), and a bias for observer (seeing oneself from the outside) perspective imagery (Williams and Moulds 2007; Nelis et al. 2013), and thus the particular emphasis in the training on using field perspective imagery and avoiding verbal analysis may be especially useful for positive outcomes in depression. However, no study to date has explored the importance of the instruction to imagine the training scenarios on clinical outcomes in individuals with depression, and none have examined whether practice in imagery use increases imagery vividness in this population.

Two studies have so far investigated the potential of imagery CBM-I in reducing symptoms of depression in clinical samples, when delivered as a stand-alone intervention. In the first such translational study, Blackwell and Holmes (2010) used a single case series design to investigate the impact of 1 week of daily sessions of imagery CBM-I in seven participants experiencing a current major depressive episode. This study demonstrated the initial promise of imagery CBM-I as a potential intervention in depression, with the group overall showing large effect sizes for improvements in depressive symptoms, interpretive bias, and general mental health at 1-week post-intervention. Depressive symptoms were also measured at a 2-week follow-up and the improvements were maintained. A main limitation of this initial study was the lack of a control group, meaning that improvement may have been attributed to non-specific aspects of the CBM-I such as distraction.

A second study therefore compared the imagery CBM-I to a control condition (Lang et al. 2012). This study used a “multi-component” CBM, comprising three sessions of the paradigm described above, two sessions of a picture-word imagery CBM-I paradigm (Holmes et al. 2008c; Pictet et al. 2011), and one session of a CBM targeting appraisals of negative intrusive memories (Lang et al. 2009). Twenty-six participants with major depression were randomly assigned to complete either 1 week of daily sessions of the positive imagery CBM-I, or a control program. In the control program half of the training stimuli were resolved positively and half were resolved negatively, thus removing the training contingency to always expect a positive resolution. Individuals receiving the positive imagery CBM-I demonstrated significant improvements from pre to post-treatment in depressive symptoms, cognitive bias, and intrusive symptoms, compared to the control condition. Improvements in depressive symptoms at 2-week follow-up were at trend level compared to the control condition. This study therefore provided further support for the clinical potential of imagery CBM-I in depression. It also demonstrated the importance of the consistently positive resolution of the training materials, rather than the effects of the program being due simply to generation of imagery per se, or non-specific effects such as distraction.

These two initial studies pave the way for larger clinical trials investigating the effects of imagery CBM-I over a longer time period. However, they also leave unanswered two key questions about the imagery component of the interventions. First, is the requirement to generate mental imagery crucial for the clinical impact of the training? In the study by Lang et al. (2012) both conditions involved generating imagery. The superiority of the positive condition suggests that it is practising *positive* imagery, rather than imagery per se, that leads to clinical improvement, despite recent research showing a general reduction in imagery vividness in depression (Torkan et al. 2012). However, this study cannot rule out the possibility that in a clinical sample of depressed individuals, simple repeated exposure to hundreds of positive-valenced stimuli may act as a positive mood induction (e.g. Velten 1968) and thus lead to improvements in symptoms of depression. We would in fact predict that without the requirement to generate imagery, people with depression would revert to a verbal, comparative, style of processing and thus fail to show improvement (cf. Holmes et al. 2009b). However, given the importance placed on generating imagery in the paradigm, the role of the instruction to use imagery is a crucial one to be tested.

Another question is whether the repeated practice in generating mental imagery leads to changes in imagery ability. This is a key question for understanding how engaging in imagery CBM-I leads to clinical benefits, as we would hypothesise that part of the helpful effects may be conferred by improvement in the ability to vividly imagine positive imagery. However, it is unclear whether imagery vividness can in fact be improved via training (Rademaker and Pearson 2012), and thus demonstrating a simple training effect on imagery vividness is a crucial first step in testing our hypothesis.

The current study therefore had three general aims. The primary aim was to investigate whether the imagery CBM-I could be successfully translated to and applied in a new, non-European and non-English-speaking, population and culture (Iran). A second aim was to investigate whether the requirement to *imagine* the positive training materials was important for the clinical impact of the training. A third aim was to investigate whether the training had any impact on the general ability to generate vivid mental imagery.

We addressed the primary aim by investigating the imagery CBM-I in a sample of treatment-seeking patients with major depression in outpatient psychiatry clinics in Iran. The initial theoretical and experimental work underpinning this imagery CBM-I approach was largely carried out in European settings, and although there are no specific theoretical reasons to expect that the approach would be ineffective in a non-European population or culture, a straightforward equivalence of effects cannot be assumed. With some exceptions (e.g. Memory Specificity Training for depression; Neshat-Doost et al. 2013) CBM interventions that showed initial promise, e.g. attention bias modification for social anxiety disorder, have sometimes struggled to translate from one language, country or platform to another (e.g. Carlbring et al. 2012). Research in mental health may be particularly sensitive to translational issues, due to cross-cultural differences in the conception and expression of psychological disorders, as well as in variation in language. In Iran there appears to be a particularly high expression of somatic symptoms in major depression (Hakimshooshtary et al. 2007), which may suggest differences in underlying processes and potential responses to treatments. For example, it has been suggested that Behavioural Activation may be a particularly effective treatment for depression in Iran due to the approach fitting well with Iranian culture (Moradveisi et al. 2013). Thus, demonstrating that an approach can survive translation from its initial place of development is an important step in developing interventions of broader potential reach. The sample in the current study represents a novel group not only in the translation to a different language and culture, but also a “real-world” sample of treatment-seeking patients. With some exceptions (e.g. Brosan et al. 2011), very few studies have investigated CBM in individuals in psychiatric settings. As a first translation to this novel population, we conducted this as a pilot study. In order to enhance comparability with published studies, we used a time-frame for the study as in the previous initial studies, namely a 1-week intervention and 2-week follow-up (Blackwell and Holmes 2010; Lang et al. 2012). We investigated the impact of the imagery CBM-I on symptoms of depression, anxiety, and negative interpretive bias (cf. Lang et al. 2012).

In order to investigate the importance of the imagery instructions, we included a control condition (“non-imagery”) in which participants were presented with an identical set of training stimuli (i.e. all positively resolved) over an identical schedule of sessions, but were given no training in imagery or instruction to imagine the scenarios. Instead they were told to listen to the scenarios and not instructed to use any particular form of processing. We further included an additional no treatment control condition in order to provide a comparison for the active control condition (cf. Watkins et al. 2009).

Finally, in order to investigate the effects of the training on mental imagery, we included at pre-treatment, post-treatment and follow-up a general measure of vividness of mental imagery in order to investigate whether the repeated practice in using mental imagery led to increased imagery vividness. We also included measures of general use of imagery and tendency to ruminate (a form of verbal processing) in everyday life in order to more fully characterise any effects of the CBM-I on imagery and verbal processing. For example, it may be that the repeated requirement to use imagery and not verbal processing within the task would generalize to a greater tendency to use mental imagery and reduced tendency to use a verbal (ruminative) processing style in everyday life. Although these are not hypothesised to be key mechanisms by which the CBM-I paradigm improves clinical outcomes, understanding the broader potential impact of the program is helpful in describing its effects more fully.

We hypothesised that:

1. The imagery CBM-I would be successfully translated to and applied in the new population, such that participants in the imagery condition would show improvements in symptoms of depression and anxiety, and reduction in negative interpretive bias over the 1-week training and at 2-week follow-up.
2. Participants in the imagery condition would show greater improvement in outcome measures than those in the non-imagery active control condition.
3. Participants in the imagery condition would show increased vividness of mental imagery following the imagery CBM-I compared to those in the control conditions.

## Method

### Overview

A mixed design was used, in which participants were randomly allocated to one of three groups: imagery-focussed positive CBM-I (imagery condition), the identical program but with no instruction to use imagery (non-imagery condition), or a no treatment control condition (no treatment condition). Following the baseline assessment, participants in the imagery condition received practice in imagery and then completed a session of imagery-focussed positive CBM-I every day from home on their home computer. Participants in the non-imagery condition completed an identical program, but without the prior instruction to use imagery or imagery practice. Participants in the no treatment condition simply returned 1 week later. Measures of depressive symptoms, anxiety, interpretive bias, use and vividness of imagery, and rumination were completed pre and post the intervention week, and in the imagery and non-imagery conditions 2 weeks later at follow-up.

### Participants

Treatment-seeking patients with major depressive disorder (MDD) were recruited from five outpatient psychiatry clinics in Isfahan, Iran. Diagnoses were determined by experienced psychiatrists based on Structured Clinical Interview for DSM-IV axis I disorders (SCID-I; First et al. 1996). Participants were recruited if they were willing to take part in a study that could involve two or four attendances over several weeks. Volunteers for participation were eligible if they met criteria for MDD (DSM-IV-TR; American Psychiatric Association 2000). Exclusion criteria were acute suicidality, depressive disorder with psychotic symptoms, history of bipolar disorder, substance-abuse disorders, organic psychiatric disorders, neurological impairment, psychosis, psychological treatment, recent change in medication and severe depression needing immediate treatment in its own right. Written informed consent was provided by all participants. MDD was the primary diagnosis in all cases. This selection process continued until 39 participants who met inclusion criteria were recruited, 13 being randomly allocated to each condition.^1^ Demographic information for the participants is presented in Table 1. Ethical approval was obtained from the University of Isfahan.

**Table 1:** Sample characteristics across conditions

	Imagery condition (*n* = 13)	Non-imagery condition (*n* = 13)	No treatment condition (*n* = 13)
Age (years), *M* (SD)	26.4 (7.82)	25.9 (7.27)	30.54 (11.2)
Gender (%)			
Female	62	77	54
Male	39	23	46
Years of education	14.9 (2.66)	13.7 (1.97)	14.6 (1.89)
Self-rated economic status (%)			
Low	8	8	8
Moderate	54	77	62
Good	39	15	31
Current medication (%)	31	23	54
Ever received treatment for depression (%)	46	39	54
Number of past episodes of depression	0.62 (0.96)	0.38 (0.65)	0.31 (0.75)
Any comorbid disorder (%)	23	15	15

Comorbid disorders were: panic disorder with agoraphobia (*n* = 1), obsessive–compulsive disorder (*n* = 3), generalized anxiety disorder (*n* = 2), dysthymic disorder (*n* = 1)

### Intervention

#### Positive Training Paragraphs

There were 448 different positive training paragraphs, which had previously been used by Blackwell and Holmes (2010). These were translated to Farsi by three English language experts in Isfahan University and finally were edited by the first author and were read in a male voice. Paragraphs lasted 7–16 s, and were digitally recorded. They were presented stereophonically through headphones. Participants were given the CBM-I programme in the form of an executable file using Adobe Flash software (CS4 Version, California; Adobe Systems Inc.), provided on a USB Flash drive. They returned this at the end of the study, allowing the researchers to verify how many sessions of the CBM-I program they had completed via the record kept by the program. The translated scenarios had the same linguistic structure as the original paragraphs, such that the positive outcome only became clear towards the end of the statement, albeit within the constraints of linguistic phrasing considerations in Farsi. For example: “You receive an essay back from your tutor and do not get the grade that you had expected. She tells you that this is because, on this occasion, your work was *outstanding*”. In translation, the resolution was still only apparent at the end of the sentences. However, we noted some slight differences in word order from British English in that the verb would typically appear at the end of a sentence in Farsi. The content of some of the scenarios was also adapted to fit into Iranian culture, for example by referring to particular events and customs: “It’s Nowrooz and your family is gathered around Haft-sin cloth. You look at them with a rush of *love and pride*” or “You have gone to a house-warming party despite being a bit under the weather. After drinking a cup of warm tea, you notice that you are beginning to feel relatively *relaxed and much better*” (resolutions in italics, change from British English was to replace ‘wine’ with ‘tea’).

Each day, participants were presented with 64 different auditory training descriptions in eight randomized blocks of eight paragraphs. All descriptions were followed by a 2 s pause. Task instruction reminders were given between blocks, with short breaks allowed between these blocks. The same training paragraphs were used in both the imagery and non-imagery condition. In order to focus participants on their assigned task (see below), after each training paragraph (and 2 s gap), participants in the imagery condition were asked to rate the vividness of their imagery (“How vividly could you imagine the situation that was described?”) as in previous studies (Blackwell and Holmes 2010; Holmes et al. 2006), whereas participants in the non-imagery condition were asked to rate continuity of listening (“How continuously could you listen to the presented description?”). Ratings were made on a 5-point scale ranging from 1 (not at all) to 5 (very). Each session started with a neutral practice item.

#### Imagery and Non-imagery Instructions

Prior to the first session of CBM-I, participants in the imagery condition were given a brief practice task in which they were asked to imagine cutting a lemon in order to clarify what is meant by “using mental imagery”. They then practiced four sample descriptions with a particular emphasis on using imagery from field perspective, and not using observer perspective imagery or verbal processing. Participants in the non-imagery condition received no training or practice, and were asked simply to listen continuously to the auditory descriptions presented via headphones. They therefore did not receive any instruction to use a particular style of processing while listening to the training scenarios.

### Outcome Measurement

#### Measures of Symptoms

##### Beck Depression Inventory-Second Edition

(BDI-II; Beck et al. 1996). The BDI-II is a well-established questionnaire measure assessing depressive symptomatology over the preceding 2 weeks. It consists of 21 items on which participants responded to a series of questions on a scale from 0 to 3 and total scores can range from 0 to 63. Scores are classified as follows: 0–13 as minimal depression; 14–19 as mild depression; 20–28 as moderate depression and 29–63 as severe depression. BDI-II total scores have generally been found to have high internal consistency (coefficient α > .90) and moderate to high convergent validities (r > .50) with other self-report and clinical rating scales of depression in psychiatric patients, college students, and normal adults (Steer and Beck 2004). The Persian version of the BDI-II with good validity and reliability (α = .91 by Dabson and Mohammadkhani 2007) was used in the current study.

##### State-Trait Anxiety Inventory

(STAI; Spielberger et al. 1983). The trait scale of the STAI was used to measure trait anxiety. This consists of 20 anxiety-related items on which participants rated how they “generally feel” on a 4-point scale: *almost never*, *sometimes*, *often*, or *always*. These widely used measures have satisfactory reliability and validity (Spielberger et al. 1983). The Persian version of the trait scale of the STAI (Panahi Shahri 1994) that was used in the current study has a good internal consistency (α = .90).

#### Measure of Negative Interpretive Bias

##### Scrambled Sentences Test

(SST; Wenzlaff 1993). The SST was used as an implicit measure of depressive interpretation bias (Phillips et al. 2010). Participants were asked to unscramble a list of 20 mixed sequence of words (e.g. winner born I am loser a) under a cognitive load (remembering a six digit number) and constrained time (4 min). It measured the tendency of participants to interpret ambiguous information either positively (I am a born winner) or negatively (I am a born loser). A “negativity” score is generated by calculating the proportion of sentences completed correctly with a negative emotional valence. For this study, this material was translated to Farsi. Rude et al. (2002) found scores on the SST to predict depressive symptoms 4–6 weeks later.

#### Measures of Imagery and Verbal Thinking Style

##### Vividness of Visual Imagery Questionnaire

(VVIQ; Marks 1973). The VVIQ is the most frequently used measure of how vividly individuals can create visual mental images. It is generally considered to be reasonably reliable and valid (McKelvie 1995). The VVIQ has 16 items in which subjects are asked to form visual images of various scenes; such as “the sun rising above the horizon into a hazy sky” (Marks 1973). Ratings range from 1 (no image at all) to 5 (image clear and vivid as a perception). Responses are summed to create a total score which higher total scores show more vivid imagery. In the current study, the questionnaire was translated into Farsi with a good internal consistency (α = .96).

##### Spontaneous Use of Imagery Scale

(SUIS; Reisberg et al. 2003). In the current study, the Persian version of SUIS (translated by the first author) with a good internal consistency (α = .83) was used to measure the trait tendency to use imagery in everyday life. This 12-item questionnaire is rated on 5-point scale ranging from 1 (never appropriate) to 5 (always completely appropriate) (e.g., “When I think about visiting a relative, I almost always have a clear mental picture of him or her”).

##### Ruminative Responses Scale

(RRS; Nolen-Hoeksema and Morrow 1991). The RRS measures how often respondents tend to ruminate in response to a sad mood. The RRS consists of 22 items that assess responses to feeling sad, down, or depressed that are focused on the self, on symptoms, and on possible causes and consequences of moods (e.g., “Why do I always react this way?”), each rated on a 4-point scale ranging from 1 (almost never) to 4 (almost always), with higher scores indicating greater tendency to ruminate (range 22–88). Previous studies using this measure have shown good test–retest reliability and acceptable convergent and predictive validity (Nolen-Hoeksema and Morrow 1991; Nolen-Hoeksema et al. 1994; Treynor et al. 2003). In the current study, the scale also was translated into Farsi and its internal consistency was good (α = .87).

### Manipulation Checks

At the post-treatment session, participants in the imagery and non-imagery conditions completed a manipulation check questionnaire asking about task difficulty (“How difficult or easy did you find your task of listening to the sentences?”, where 1 = extremely difficult and 9 = extremely easy), use of imagery (“How much did you find yourself thinking in IMAGES (i.e. in mental pictures and sensory impressions) as you were listening to the sentences?”, where 1 = not at all and 9 = all the time), use of verbal analysis (“How much did you find yourself VERBALLY ANALYSING THE MEANING of the sentences as you were listening to them?”, where 1 = not at all and 9 = all the time), and difficulty maintaining focus (“How much of the time did you find it difficult did you find it to focus on your task, i.e. find it difficult to concentrate and that your attention wandered?”, where 1 = not at all and 9 = all the time). They were then interviewed in more detail about their use of imagery or verbal processing (cf. Blackwell and Holmes 2010). Participants in the non-imagery condition had the different modes of processing (e.g. imagery vs. verbal) explained to them prior to completing the manipulation checks, as this had not been explained to them previously.

### Procedure

After providing written informed consent at the initial assessment session, participants were randomly assigned to the imagery, non-imagery or no treatment conditions. Participants in the no treatment condition completed the outcome measures (pre-treatment) and returned to the psychiatry clinics 1 week later to repeat them (post-treatment). They were then referred on for treatment. Following the initial assessment session, participants in the imagery and non-imagery conditions completed the outcome measures (pre-treatment), followed by a first session of the relevant CBM-I program. In the imagery condition, this session included the imagery practice described above. Over the subsequent week participants completed a session of the CBM-I program every day from home. At the end of this intervention week they returned to the psychiatry clinics to repeat the outcome measures (post-treatment) and manipulation check questionnaire, and were interviewed about their experience of completing the corresponding CBM-I program. They returned to the psychiatry clinics 2 weeks later to repeat the outcome measures (follow-up). All of the assessments and the first session of CBM-I were completed individually at one of the psychiatry clinics.

## Results

### Randomization Checks

The three groups did not statistically differ with regard to demographic or clinical characteristics, or any of the baseline measures (all *p*s > .2; see Tables 1, 2). All participants completed the post-treatment assessment, and there was no significant difference between rate of attrition to follow-up following randomization to imagery or non-imagery condition, 38.5 versus 38.5 %; χ2 (1, *n* = 26) < 0.01, *p* = 1.000. Reasons for drop-out from post-treatment to follow-up were, in the imagery condition: Moving out of the area (*n* = 2), not able to attend due to exams starting (*n* = 2), starting treatment for depression (*n* = 1), and in the non-imagery condition: Starting treatment for depression (*n* = 2), family crisis (*n* = 1), did not wish to attend the assessment (*n* = 2).

**Table 2:** Outcome measures at pre-treatment, post-treatment, and follow-up across conditions

	Pre-treatment		Post-treatment (1 week after pre-treatment)		Follow-up (2 weeks after post-treatment)	
	*M*	SD	*M*	SD	*M*	SD
BDI-II						
Imagery condition	33.62	7.89	21.15	10.11	16.50	5.50
Non-imagery condition	29.62	9.33	25.69	11.33	25.50	5.73
No treatment condition	29.54	6.98	26.92	11.49	–	–
STAI-T						
Imagery condition	61.23	6.69	54.46	9.08	55.38	8.35
Non-imagery condition	59.23	7.98	57.77	11.66	57.25	10.43
No treatment condition	58.23	7.54	55.54	9.48	–	–
SST Negativity						
Imagery condition	47.25	23.97	26.02	16.80	15.02	17.09
Non-imagery condition	37.56	20.46	44.76	20.36	49.62	19.46
No treatment condition	35.33	23.66	39.09	14.69	–	–
VVIQ						
Imagery condition	37.85	17.21	46.85	12.46	48.63	15.44
Non-imagery condition	43.62	13.74	44.38	15.68	43.50	20.45
No treatment condition	46.31	11.22	44.31	13.92	–	–
SUIS						
Imagery condition	40.23	9.22	42.92	8.69	41.50	8.25
Non-imagery condition	39.00	8.44	37.23	10.35	40.88	12.55
No treatment condition	39.85	8.24	40.15	6.62	–	–
RRS						
Imagery condition	63.46	11.35	44.38	11.81	43.00	8.35
Non-imagery condition	57.23	6.69	53.92	15.71	53.88	13.90
No treatment condition	60.62	11.81	58.54	9.49	–	–

*BDI*-*II* Beck Depression Inventory-II, *STAI*-*T* trait subscale of the State–Trait Anxiety Inventory, *SST Negativity* scrambled sentences test (percentage of sentences completed negatively), *VVIQ* Vividness of Visual Imagery Questionnaire; *SUIS* Spontaneous Use of Imagery Scale; *RRS* Ruminative Responses Scale

Of participants assigned to the imagery or non-imagery conditions, all completed at least six of the possible seven sessions. There was no significant difference between imagery condition (*M* = 6.54, SD = 0.66) and non-imagery condition (*M* = 6.38, SD = 0.96) on the number of CBM-I sessions completed, *t*(24) < 1. Participants were included in the analyses regardless of how many sessions they had completed.

### Manipulation Check and Debriefing

Participants rated the task as equally easy in both the imagery (*M* = 5.54, SD = 2.70) and non-imagery (*M* = 5.85, SD = 2.48) conditions, *t*(24) < 1. Participants in the imagery condition reported more use of imagery than those in the non-imagery condition (*M* = 7.23, SD = 1.24 vs. *M* = 5.31, SD = 2.02; *t*(24) = 2.93, *p* = .007). Participants in the imagery condition reported less use of verbal analysis than those in the non-imagery condition (*M* = 3.69, SD = 1.60 vs. *M* = 6.54, SD = 1.80; *t*(24) = 4.25, *p* < .001). Participants rated maintaining focus as equally difficult in both the imagery (*M* = 4.08, SD = 1.80) and non-imagery (*M* = 3.92, SD = 1.98) conditions, *t*(24) < 1. Information gathered during the debriefing interview further suggested that participants in the imagery condition had predominantly used field perspective, rather than observer perspective, imagery; conversely within the non-imagery condition all but one participant reported that when they did use imagery it had been predominantly observer rather than field perspective. Thus it appeared that the condition manipulation had been successful in leading to different styles of processing in the two CBM-I conditions. Participants in the imagery condition, and hence engaging in more imagery and less verbal processing, were more likely to describe the sessions as enjoyable. This is consistent with the feedback from a previous study that participants who struggled to engage in imagery and used a more verbal processing style tended to find the sessions more tedious (Blackwell and Holmes 2010).

### Post-Treatment Outcome Analysis

#### Measures of Symptoms

##### Depressive Symptoms

For the BDI-II there was a significant main effect of time, *F*(1,36) = 22.14, *p* < .001, η^2^ = .38, but not of condition, *F*(2,36) < 1. There was a significant interaction of time with condition, *F*(2,36) = 5.26, *p* = .010, η^2^ = .23. There was a significant decrease from pre to post-treatment within both the imagery condition, *M* = 12.46, SD = 9.23, *t*(12) = 4.87, *p* < .001, *d* = 1.58, and within the non-imagery condition, *M* = 3.92, SD = 5.78, *t*(12) = 2.45, *p* = .031, *d* = 0.42, but no decrease within the no treatment condition, *M* = 2.62, SD = 9.66, *t*(12) < 1. The reduction in depressive symptoms in the imagery condition was significantly greater than that in both the non-imagery, *t*(24) = 2.82, *p* = .009, *d* = 1.11, and the no treatment condition, *t*(24) = 2.66, *p* = .014, *d* = 1.04. The reduction in depressive symptoms in the non-imagery condition was not significantly different from that in the no treatment condition, *t*(24) < 1.

##### Trait Anxiety

For the STAI-T there was a significant main effect of time, *F*(1,36) = 8.17, *p* = .007, η^2^ = .19, but not of condition, *F*(2,36) < 1, and there was no significant interaction of time with condition, *F*(2,36) = 1.59, *p* = .219.

##### Measure of Negative Cognitive Bias

For the SST, there was no main effect of time, *F*(1,36) < 1, and no significant effect of condition, *F*(2,36) < 1, but there was a significant interaction of time with condition, *F*(2,36) = 6.82, *p* = .003, η^2^ = .28. There was a significant decrease from pre to post-treatment within the imagery condition, *M* = 21.23, SD = 20.62, *t*(12) = 3.71, *p* .003, *d* = 0.89, but not within the non-imagery condition, *M* = 7.20 (increase), SD = 15.39, *t*(12) = 1.69, *p* = .117, nor within the no treatment condition, *M* = 3.75 (increase), SD = 26.76, *t*(12) < 1. The reduction in negative interpretive bias in the imagery condition was significantly greater than that in both the non-imagery, *t*(24) = 3.99, *p* = .001, *d* = 0.77, and the no treatment condition, *t*(24) = 2.67, *p* = .013, *d* = 0.73. There was no significant difference in change between the non-imagery and no treatment condition, *t*(24) < 1.

#### Measures of Imagery and Ruminative Thinking Style

##### Vividness of Visual Imagery

For the VVIQ there were no significant main effects of time, *F*(1,36) = 2.66, *p* = .112 or condition, *F*(2,36) < 1. However, there was a significant interaction of time with condition, *F*(2,36) = 4.32, *p* = .021, η^2^ = .19. There was a significant increase from pre to post-treatment within the imagery condition, *M* = 9.00, SD = 13.18, *t*(12) = 2.46, *p* = .030, *d* = 0.52, but not within the non-imagery condition, *M* = 0.77, SD = 8.72, *t*(12) < 1, nor within the no treatment condition, *M* = 2.00 (decrease), SD = 6.77, *t*(12) = 1.07, *p* = .308. The increase in vividness of visual imagery in the imagery condition was at trend level compared to the non-imagery condition, *t*(24) = 1.88, *p* = .073, *d* = 0.73, and significantly greater than that in the no treatment condition, *t*(24) = 2.68, *p* = .013, *d* = 0.67. There was no significant difference in change between the non-imagery and no treatment condition, *t*(24) < 1.

##### General Use of Imagery

For the SUIS there were no significant main effects of time, *F*(1,36) < 1 or condition, *F*(2,36) < 1, and no significant interaction of time with condition, *F*(2,36) = 2.28, *p* = .117.

##### Rumination

For the RRS there was a significant main effect of time, *F*(1,36) = 11.89, *p* = .001, but not of condition, *F*(2,36) = 1.42, *p* = .254. There was a significant interaction of time with condition, *F*(2,36) = 5.36, *p* = .009, η^2^ = .23. There was a significant decrease in ruminative responses from pre to post-treatment within the imagery condition, *M* = 19.08, SD = 17.12, *t*(12) = 4.02, *p* = .002, *d* = 1.68, but not within the non-imagery condition, *M* = 3.31, SD = 14.22, *t*(12) < 1, or within the no treatment condition, *M* = 2.08, SD = 12.60, *t*(12) < 1. The decrease in ruminative responses in the imagery condition was significantly greater than that in both the non-imagery condition, *t*(24) = 2.56, *p* = .017, *d* = 1.00, and that in the no treatment condition, *t*(24) = 2.88, *p* = .008, *d* = 1.13. There was no significant difference in change between the non-imagery and no treatment condition, *t*(24) < 1.

### Follow-up

The follow-up analyses compare only the imagery and non-imagery conditions, as participants in the no treatment condition were not requested to return for a follow-up. Only those participants who provided follow-up data (*n* = 8 in the imagery condition, and *n* = 8 in the non-imagery condition) are included in these analyses.

#### Measures of Symptoms

##### Depressive Symptoms

For the BDI-II there was a significant main effect of time, *F*(1,14) = 26.14, *p* < .001, η^2^ = .65, but not of condition, *F*(1,14) < 1. There was a significant interaction of time with condition, *F*(1,14) = 12.26, *p* = .004, η^2^ = .47. There was a significant decrease from pre-treatment to follow-up within the imagery condition, *M* = 17.38, SD = 7.82, *t*(7) = 6.29, *p* < .001, *d* = 2.17, but not within the non-imagery condition, *M* = 3.25, SD = 8.31, *t*(7) = 1.11, *p* = .305. The reduction in depressive symptoms in the imagery condition was significantly greater than that in the non-imagery condition, *t*(14) = 3.50, *p* = .004, *d* = 1.75.

##### Trait Anxiety

For the STAI there was a significant main effect of time, *F*(1,14) = 7.41 *p* = .017, η^2^ = .35, but not of condition, *F*(1,14) < 1. There was a significant interaction of time with condition, *F*(1,14) = 5.06, *p* = .041, η^2^ = .27. There was a significant decrease from pre-treatment to follow-up within the imagery condition, *M* = 7.88, SD = 5.17, *t*(7) = 4.31, *p* = .004, *d* = 0.87, but not within the non-imagery condition, *M* = 0.75, SD = 7.32, *t*(7) < 1. The reduction in trait anxiety in the imagery condition was significantly greater than that in the non-imagery condition, *t*(14) = 2.25, *p* = .041, *d* = 1.13.

#### Measure of Negative Cognitive Bias

For the SST there was no significant main effect of time, *F*(1,14) = 1.06, *p* = .321, or condition, *F*(1,14) = 2.40, *p* = .143. However, there was a significant interaction of time with condition, *F*(1,14) = 21.57, *p* < .001, η^2^ = .61. There was a significant decrease from pre-treatment to follow-up within the imagery condition, *M* = 25.76, SD = 24.46, *t*(7) = 2.98, *p* = .021, *d* = 1.34, but a significant increase within the non-imagery condition, *M* = 16.41, SD = 7.82, *t*(7) = 5.93, *p* = .001, *d* = 0.59. The reduction in negative interpretive bias in the imagery condition was significantly greater than that in the non-imagery condition, *t*(14) = 4.65, *p* < .001, *d* = 2.32.

#### Measures of Imagery and Ruminative Thinking Style

##### Vividness of Visual Imagery

For the VVIQ there was a trend level effect of time, *F*(1,14) = 3.51, *p* = .082, *d* = 0.20, but no significant effect of condition, *F*(1,14) < 1. There was a significant interaction of time with condition, *F*(1,14) = 7.58, *p* = .016, η^2^ = .35. There was an increase from pre-treatment to follow-up within the imagery condition, *M* = 10.75, SD = 9.77, *t*(7) = 2.53, *p* = .039, *d* = 0.63, but not within the non-imagery condition, *M* = 2.50, SD = 6.52, *t*(7) = 1.08, *p* = .314. The increase in vividness of visual imagery in the imagery condition was significantly greater than that in the non-imagery condition, *t*(14) = 2.75, *p* = .016, *d* = 0.99.

##### General Use of Imagery

For the SUIS there were no significant main effects of time, *F*(1,14) < 1 or condition, *F*(1,14) < 1, and no significant interaction of time with condition, *F*(1,14) = 1.76, *p* = .205.

##### Rumination

For the RRS there was a significant main effect of time, *F*(1,14) = 7.20, *p* = .018, η^2^ = .34 but not of condition, *F*(1,14) = 1.83, *p* = .198. The interaction of time with condition was not significant, *F*(1,14) = 2.57, *p* = .131.

#### Further Follow-up Analyses

In response to helpful reviewer suggestions, to further understand where the clinical change occurred, we carried out a similar ANOVA examining the change from post-treatment to follow-up for the BDI-II. There was no significant main effect of time, *F*(1,14) = 1.05, *p* = .324, and no significant interaction between time and condition, *F*(1, 14) < 1, suggesting that the improvement in symptoms of depression occurred during the week of training and was maintained at follow-up. An ANOVA examining change from post-treatment to follow-up for the STAI showed no significant effects of time, condition, or their interaction (all *F*s < 1). For the RRS, such an ANOVA showed no significant effect of time or time by condition interaction (*F*s < 1), but there was a significant main effect of condition, *F*(1, 14) = 6.66, *p* = .022, η^2^ = .32.

There was no difference between participants who did or not drop out at follow-up in terms of age, gender, or baseline depression severity (BDI-II score at pre-treatment), within either the imagery or within the non-imagery condition (all *p*s > .1).

### Analysis of Clinically Significant Change on the BDI-II

Clinically significant change was defined as a shift to a lower category of depressive symptom severity accompanied by a reduction greater than the reliable change index of 7.16, calculated according to the guidance provided by Jacobson and Truax (1991) and as applied in previous studies (Blackwell and Holmes 2010; Lang et al. 2012). In the imagery condition, 69.2 % of participants demonstrated clinically significant change over the 1-week intervention, compared to 23.1 % in the non-imagery condition and 46.2 % in the no treatment condition. The difference between the three percentages was at trend level, χ^2^ (2, 39) = 5.57, *p* = .062. Further exploration demonstrated that significantly more participants showed clinically significant change over the 1-week intervention in the imagery condition compared to the non-imagery condition, *p* = .047, Fisher’s exact test, but not compared to the no treatment condition, *p* = .428, Fisher’s exact test. There was no significant difference in rates of clinically significant change between the non-imagery and no-treatment conditions, *p* = .41, Fisher’s exact test. From pre-treatment to follow-up, significantly more participants in the imagery condition demonstrated clinically significant change compared to those in the non-imagery condition, 87.5 versus 12.5 %, *p* = .010, Fisher’s exact test.

### Relationship Between Change in Symptoms of Depression, Bias, and Imagery

To investigate whether changes in symptoms of depression from pre to post-treatment were related to the potential mechanisms of change targeted by the CBM-I (cognitive bias, imagery vividness) we carried out correlations between the relevant change scores in the whole sample. There was a significant correlation between reduction in BDI-II over the 1 week from pre to post-treatment and both reduction in negativity score on the SST (*r*(37) = .33, *p* = .042), and increase in score on the VVIQ (*r*(37) = .39, *p* = .014) over this week.

### Additional Post-Hoc Analyses

One potential concern with small samples is that analyses of means can sometimes be unreliable, as the means may be disproportionately influenced by outliers. We therefore examined our data for the main outcome (change in BDI-II from pre to post-treatment) on an individual-level basis. The pattern of individual level change on the BDI-II from pre to post-treatment showed a high level of consistency, demonstrative of systematic differences between the groups in their response rather than sampling bias, increasing our confidence in the reliability of our main statistical analyses. As a graphic example of this, all (*n* = 10, 76 %) but 3 of the positive imagery group were clustered at the top end of the “responders” in terms of change in BDI–II from pre to post-intervention, compared to only 15 % (*n* = 2) of the active control group or 30 % (*n* = 4) of the no treatment group in this range (top 16).

## Discussion

The present pilot study is, to our knowledge, the first investigation of imagery CBM-I for depression in a non-European, non-English-speaking country, the first to investigate the importance of the use of imagery instructions in the clinical impact of repeated sessions of this paradigm, and the first to investigate whether the training can increase the vividness of imagination in depressed individuals. In a sample of patients presenting with major depression to psychiatric outpatient clinics in Iran, compared to two control conditions we found that engaging in repeated sessions of imagery CBM-I over the course of 1 week reduced symptoms of depression and negative interpretive bias at 1-week post-treatment and 2-week follow-up. A significant reduction in trait anxiety was also found at 2-week follow-up. Further, we found evidence supportive of the importance of the instruction to imagine the training scenarios in the clinical impact of the training. In fact, participants simply instructed to listen to and focus on the scenarios showed no more improvement in symptoms of depression or other outcomes than a no treatment control group. Finally, we found that repeated practice in generating imagery resulted in increases in general (non-emotional) mental imagery ability, as measured by self-reported imagery vividness. This study therefore provides preliminary evidence for the potential cross-cultural applicability of a positive imagery training paradigm in depression, and furthers our knowledge of the parameters and effects of this form of training. The implications of the current study and how it builds on previous work will be discussed first in relation to the clinical outcomes, then the role of imagery in the training, and finally the impact of the training on imagery.

The success of the imagery CBM-I in the current study when translated to a new setting and population is encouraging, and supplements the initial findings from other preliminary studies to date (Blackwell and Holmes 2010; Lang et al. 2012). Consistent with these studies, which also used a schedule of 1 week of daily imagery CBM-I and a 2-week follow-up, significant reductions in symptoms of depression and negative cognitive bias were found over the 1-week intervention (*n* = 13 per group) and subsequent 2 week follow-up (*n* = 8 per group), corresponding to large effect sizes. Rates of clinically significant change in symptoms of depression were generally high in this study in comparison to previous studies. For example, in the imagery condition, the percentage of participants showing clinically significant change were 69.2 and 87.5 % from pre to post-treatment and pre-treatment to follow-up respectively, whereas these figures were 46.2 and 53.8 % in the study by Lang et al. (2012). The high rate of clinically significant change in the no treatment condition over the 1 week intervention period (46.2 %) is surprising, and it was not significantly different from that found in either the imagery or non-imagery condition. The emergence of the reduction in trait anxiety only at follow-up within the imagery condition in the current study could be due to the relative insensitivity to change of a trait scale, or it could be that changes in anxiety only emerged over a longer time period, once participants had had sufficient experience of deploying the newly trained bias in their daily lives, as has occurred in some other training studies (e.g. Browning et al. 2012).

The demonstration in the current study of the importance of the imagery instructions is an important translational step that builds on earlier experimental studies (Holmes et al. 2006; Holmes et al. 2008a, 2009b). In the current study, participants in the imagery condition were instructed to imagine themselves in the training scenarios, and participants in the non-imagery condition were not instructed to use any particular mode of processing. However, participants in both imagery and non-imagery conditions listened to the same positive training scenarios. It may at first seem surprising that positive information alone, that is, spending approximately 20 min every day for 1 week listening (with no imagery instructions) to hundreds of miniature stories with positive endings had no more impact on mood than engaging in no intervention whatsoever. However, this is consistent with our knowledge of the natural processing style observed in depression, and experimental studies comparing imagery to verbal processing.

In the current study, in the absence of instruction to use a particular mode of processing, participants in our non-imagery condition appeared to process the positive training materials in the verbal, ruminative style that characterises depression (Koster et al. 2011), and may contribute to difficulties in using positive memories to improve mood (Werner-Seidler and Moulds 2012; Joormann et al. 2007). Experimental studies in non-clinical samples have demonstrated that verbal processing of positive material does not improve mood or bias, and can even lead to deterioration of mood over a single session of positive CBM-I (Holmes et al. 2006, Holmes et al. 2008a, 2009b). We might therefore expect that, in the absence of instructions to use imagery, depressed individuals might use their natural (verbal) processing style and thus fail to gain any benefit from the repeated sessions of CBM-I. However, the importance of the imagery instructions on clinical outcomes over repeated sessions of positive CBM-I in a depressed sample had hitherto not yet been investigated. This study therefore extends the implications of the earlier experimental work to a clinical population, and complements the previous clinical study by Lang et al. (2012). While the study by Lang et al. (2012) demonstrated the importance of the consistently positive resolutions of the training material to be imagined, the current study demonstrated the importance of being required to *imagine* the consistently positive training materials. Taking together the results from this study with those from the study by Lang et al. (2012), we now have some initial evidence that it may be the *combination* of the use of mental imagery with the consistently positive resolution of the training stimuli that accounts for the clinical impact of the imagery CBM-I paradigm on symptoms of depression, rather than either aspect of the training in isolation (cf. Hirsch et al. 2006; Holmes et al. 2009a).

While previous studies (Blackwell and Holmes 2010; Lang et al. 2012) have demonstrated effects of the imagery CBM-I on symptoms of depression and cognitive bias, this study is the first to our knowledge to provide evidence that engaging in repeated practice in generating mental images over the course of the CBM-I intervention could result in improvements in mental imagery ability, specifically increased vividness of visual mental imagery. This is potentially important, as depression is associated with reduced imagery vividness (Torkan et al. 2012), and in particular with reduced vividness for positive future events (Morina et al. 2011). At the other end of the spectrum, optimism, which may be seen as the polar opposite of the pessimistic thinking style associated with depression, is associated with increased vividness of positive future imagery (Blackwell et al. 2013). As being unable to imagine positive events in the future may contribute to depressed mood, increasing the vividness of this imagery may have useful clinical benefits in depression.

Although our measure of imagery vividness, the VVIQ, is a general measure of imagery vividness rather than of positive imagery, demonstrating that imagery vividness can be improved via repeated practice is an important step in understanding the potential mechanisms by which imagery CBM-I may have a therapeutic impact in depression. Interestingly, participants in the imagery condition did not show a significant increase in their score on the SUIS (Reisberg et al. 2003). Thus, the repeated practice in using imagery during the training sessions did not appear to generalize to a tendency to use (non-emotional) everyday imagery more outside of the training sessions. This is perhaps unsurprising and it was not an a priori hypothesis that this would increase; rather we were keen to determine which (of the many) aspects of imagery would be influenced during the training. It is worth noting that our results are consistent with the suggestion that the quality of imagery (vividness) may be a more important target for intervention in depression than frequency of use of (non-emotional) imagery per se. The tendency to use imagery may not in itself be adaptive or maladaptive, as it can amplify the affective impact of both negative (Holmes and Mathews 2005) and positive (Holmes et al. 2006) information, and in fact inducing a more image-based (concrete) mode of processing during a success experience does not result in greater improvement in affect compared to a more verbal (abstract) mode of processing in dysphoric individuals (Hetherington and Moulds 2013). Therefore specifically improving imagery vividness, rather than encouraging frequency of imagery use more generally, may be a useful aspect of this CBM-I. This will be interesting to explore further in larger studies and perhaps with better measures of imagery (cf. Pearson et al. 2013).

The results of the current study must be interpreted in the context of several limitations. It is important to bear the sample size (*n* = 13 per group at post-treatment and *n* = 8 per group at follow-up) in mind when interpreting the results from this study. As a first attempt (to our knowledge) to implement imagery CBM-I in this novel population, a small pilot study was appropriate in order to provide the initial evidence that could justify the time, resources, and participant burden of larger clinical trial in this new setting. However, this small sample size means that the results can only be interpreted as providing encouraging preliminary evidence. The results from pre-treatment to follow-up in particular must be interpreted with caution, due to the lack of outcome measurement in the no treatment group at follow-up, and the attrition in the imagery and non-imagery groups at this time point. The high rate of attrition at follow-up was mostly due to practical difficulties in attending another face-to-face assessment session, and thus future studies could enhance collection of follow-up data by having this data completed remotely, e.g. via online questionnaires or phone interviews. One concern when such promising clinical results are obtained with a small study relates to whether they can be replicated, as a small sample limits the generalizability of a study’s findings. We note that the results of this study are consistent with the two previous clinical studies (Blackwell and Holmes 2010; Lang et al. 2012), the experimental studies that preceded these (Holmes et al. 2006; Holmes et al. 2008a, 2009b), and a later randomized controlled trial (Williams et al. 2013). As the current study forms part of such a series of experiments, we can be more confident in the potential replicability of the results than if it was one promising study in isolation. Initial clinical translation studies like the current pilot study are not intended to provide conclusive proofs, but rather form an important step in a treatment development process and must be interpreted in this context. Some further cautions related to the sample size are noted below in discussion of several specific analyses.

The correlational analyses must be interpreted with caution due to the sample size, which limits statistical analysis of the mechanisms of change. However, the correlations suggest that for the sample as a whole, reduction in symptoms of depression was related both to reduction in negative interpretive bias, and to increase in imagery vividness over the 1 week from pre to post-treatment. Although it is not possible to draw conclusions about causal inference from these correlational data, they are at least consistent with the argument that the greater the extent to which whatever intervention (or no intervention) the participants engaged in modified their interpretive bias or imagery vividness, the more they experienced a reduction in symptoms of depression. The relationship between the putative active mechanisms of change in this CBM-I, imagery and interpretation, and clinical outcomes will be important to investigate more fully in larger samples and over longer time periods. It will also be important to investigate the potential interaction between the cognitive biases targeted (e.g. Everaert et al. 2012; Hirsch et al. 2006; Salemink et al. 2010; Tran et al. 2011). For example, studies in healthy volunteers (e.g. Holmes et al. 2006) have demonstrated that a single session of imagery CBM-I has an immediate impact on training a more positive bias and increasing positive mood. In order to further enhance the clinical potential of the CBM-I paradigm it will be useful to investigate the relative impact of these immediate effects of training sessions (e.g. change in bias, transient increase in positive mood) on longer-term clinical outcomes. The significant increase in one of the six outcome measures (scrambled sentences test) from pre-treatment to follow-up within the non-imagery condition would also need further investigation in larger samples.

Mental imagery represents a number of complex cognitive processes and plays numerous roles in daily functioning (Holmes and Mathews 2010). It will be useful for future studies to more fully characterise the impact on various aspects of mental imagery by using a more comprehensive range of measures (cf. Pearson et al. 2013), and imagery-based measures of cognitive bias (Berna et al. 2011). For example, as self-report measures of imagery such as used in this study may be subject to demand, it will be useful to investigate the effects of imagery interventions on performance-based measures of imagery ability. Further, it will be important to investigate whether the training has a differential impact on positive and negative imagery, and whether the fact that participants are required to generate field perspective imagery reduces the bias in depression to take an observer perspective (Nelis et al. 2013; Williams and Moulds 2007). Finally, it is important to note that another potential explanation for the superiority of the imagery condition in this study is that it required active generation of the positive outcomes, in the form of mental images, and it was this active generation rather than imagery per se that was the crucial difference between the two active conditions (cf. Hoppitt et al. 2010).

The development and dissemination of novel treatment approaches to help tackle the global health problem presented by depression requires that novel interventions translate from one country to another. This pilot study provides some preliminary evidence that the benefits of training positive imagination could possibly transcend national and cultural boundaries and encourages further investigation of the application of imagery CBM-I in novel populations. Further, it indicates the potential importance of mental imagery in translating the positive resolutions of the training scenarios into positive clinical outcomes in depression.

## Footnotes

1 This small sample size is congruent with the study’s aims as a pilot study. We also note that the study could be considered as adequately powered to meet its primary aim on the basis of a formal power calculation using an effect size estimate derived from the most comparable previous study. The main aim of the current study was to evaluate the impact of the imagery CBM-I on symptoms of depression relative to the control groups. This is determined by the condition by time interaction in the ANOVA investigating change in scores on the BDI from pre to post-treatment. In the most comparable published study (Lang et al. 2012), the effect size for the equivalent interaction was large (*η* ^2^ = .19). We might therefore plan to look for a large interaction effect in the current study. A power calculation (G*Power 3.1.7; Faul et al. 2007) to estimate the sample size needed to provide 80 % power to find a large effect size of *η* ^2^ = .14 (i.e. the lowest boundary of a large effect size), *α* = .05, and using the most conservative estimate of correlation between repeated measures, *r* = 0, suggests that a total sample size *N* = 36, or *n* = 12 per group, would be needed.

## References

[CR1] American Psychiatric Association (2000). Diagnostic and statistical manual of mental disorders. Text revision.

[CR2] Beck AT, Steer RA, Brown GK (1996). The Beck depression inventory.

[CR3] Berna C, Lang TJ, Goodwin GM, Holmes EA (2011). Developing a measure of interpretation bias for depressed mood: An ambiguous scenarios test. Personality and Individual Differences.

[CR4] Blackwell SE, Holmes EA (2010). Modifying interpretation and imagination in clinical depression: A single case series using cognitive bias modification. Applied Cognitive Psychology.

[CR5] Blackwell SE, Rius-Ottenheim N, Schulte-van Maaren YWM, Carlier IVE, Middlekoop VD, Zitman FG, Spinhoven P, Holmes EA, Giltay EJ (2013). Optimism and mental imagery: A possible cognitive marker to promote wellbeing?. Psychiatry Research.

[CR6] Brosan L, Hoppitt L, Shelfer L, Sillence A, Mackintosh B (2011). Cognitive bias modification for attention and interpretation reduces trait and state anxiety in anxious patients referred to an out-patient service: Results from a pilot study. Journal of Behavior Therapy and Experimental Psychiatry.

[CR7] Browning M, Holmes EA, Charles M, Cowen PJ, Harmer CJ (2012). Using attentional bias modification as a cognitive vaccine against depression. Biological Psychiatry.

[CR8] Butler G, Mathews A (1983). Cognitive processes in anxiety. Advances in Behaviour Research and Therapy.

[CR9] Carlbring P, Apelstrand M, Sehlin H, Amir N, Rousseau A, Hofmann SG, Andersson G (2012). Internet-delivered attention bias modification training in individuals with social anxiety disorder—a double blind randomized controlled trial. BMC Psychiatry.

[CR10] Dabson K, Mohammadkhani P (2007). Psychometric characteristics of Beck depression inventory-II in patients with major depressive disorder. Journal of Rehabilitation.

[CR11] Everaert J, Koster EH, Derakshan N (2012). The combined cognitive bias hypothesis in depression. Clinical Psychology Review.

[CR100] Faul, F., Erdfelder, E., Lang, A-G, & Buchner, A. (2007). G*Power 3: A flexible statistical power analysis program for the social, behavioral, and biomedical sciences. *Behavior Research Methods*, *39*, 175–191. 10.3758/bf03193146 17695343

[CR12] First MB, Spitzer RL, Gibbon M, Williams JB (1996). User’s guide for the structured clinical interview for DSM-IV axis I disorders.

[CR13] Gotlib IH, Joormann J (2010). Cognition and depression: Current status and future directions. Annual Review of Clinical Psychology.

[CR14] Hakimshooshtary M, Alagheband Rad J, Sharifi V, Arabgol F, Shabani A, Shahrivar Z (2007). Profile of major depressive disorder symptoms among patients in Tehran. Iranian Journal of Psychiatry.

[CR15] Hertel PT, Mathews A (2011). Cognitive bias modification: Past perspectives, current findings, and future applications. Perspectives on Psychological Science.

[CR16] Hetherington K, Moulds ML (2013). Does mode of processing during a positive experience have consequences for affect?. Journal of Behavior Therapy and Experimental Psychiatr.

[CR17] Hirsch CR, Clark DM, Mathews A (2006). Imagery and interpretations in social phobia: Support for the combined cognitive biases hypothesis. Behavior Therapy.

[CR18] Holmes EA, Coughtrey AE, Connor A (2008). Looking through or at rose-tinted glasses? Imagery perspective and positive mood. Emotion.

[CR20] Holmes EA, Lang TJ, Moulds ML, Steele AM (2008). Prospective and positive mental imagery deficits in dysphoria. Behaviour Research and Therapy.

[CR19] Holmes EA, Lang TJ, Deeprose C (2009). Mental imagery and emotion in treatment across disorders: Using the example of depression. Cognitive Behaviour Therapy.

[CR21] Holmes EA, Lang TJ, Shah DM (2009). Developing interpretation bias modification as a ‘cognitive vaccine’ for depressed mood: Imagining positive events makes you feel better than thinking about them verbally. Journal of Abnormal Psychology.

[CR22] Holmes EA, Mathews A (2005). Mental imagery and emotion: A special relationship?. Emotion.

[CR23] Holmes EA, Mathews A (2010). Mental imagery in emotion and emotional disorders. Clinical Psychology Review.

[CR24] Holmes EA, Mathews A, Dalgleish T, Mackintosh B (2006). Positive interpretation training: Effects of mental imagery versus verbal training on positive mood. Behavior Therapy.

[CR25] Holmes EA, Mathews A, Mackintosh B, Dalgleish T (2008). The causal effect of mental imagery on emotion assessed using picture-word cues. Emotion.

[CR26] Hoppitt L, Mathews A, Yiend J, Mackintosh B (2010). Cognitive bias modification: The role of active training in modifying emotional responses. Behavior Therapy.

[CR27] Jacobson NS, Truax P (1991). Clinical significance: A statistical approach to defining meaningful change in psychotherapy research. Journal of Consulting and Clinical Psychology.

[CR28] Joormann J, Siemer M, Gotlib IH (2007). Mood regulation in depression: Differential effects of distraction and recall of happy memories on sad mood. Journal of Abnormal Psychology.

[CR29] Koster EHW, De Lissnyder E, Derakshan N, De Raedt R (2011). Understanding depressive rumination from a cognitive science perspective: The impaired disengagement hypothesis. Clinical Psychology Review.

[CR30] Lang TJ, Blackwell SE, Harmer C, Davison P, Holmes EA (2012). Cognitive bias modification using mental imagery for depression: Developing a novel computerized intervention to change negative thinking styles. European Journal of Personality.

[CR31] Lang TJ, Moulds ML, Holmes EA (2009). Reducing depressive intrusions via a computerized cognitive bias modification of appraisals task: Developing a cognitive vaccine. Behaviour Research and Therapy.

[CR32] MacLeod C, Koster EHW, Fox E (2009). Whither cognitive bias modification research? Commentary on the special section articles. Journal of Abnormal Psychology.

[CR33] MacLeod C, Mathews A (2012). Cognitive bias modification approaches to anxiety. Annual Review of Clinical Psychology.

[CR34] Marks DF (1973). Visual imagery differences in the recall of pictures. British Journal of Psychology.

[CR35] Mathews A, Mackintosh B (2000). Induced emotional interpretation bias and anxiety. Journal of Abnormal Psychology.

[CR36] Mathews A, MacLeod C (2005). Cognitive vulnerability to emotional disorder. Annual Review of Clinical Psychology.

[CR37] McKelvie SJ (1995). Vividness of visual imagery: Measurement, nature, function, and dynamics. Journal of Mental Imagery Series.

[CR38] Moradveisi L, Huibers MJH, Renner F, Arasteh M, Arntz A (2013). Behavioural activation v. antidepressant medication for treating depression in Iran: Randomised trial. British Journal of Psychiatry.

[CR39] Morina N, Deeprose C, Pusowski C, Schmid M, Holmes EA (2011). Prospective mental imagery in patients with major depressive disorder or anxiety disorders. Journal of Anxiety Disorders.

[CR40] Nelis S, Debeer E, Holmes EA, Raes F (2013). Dysphoric students show higher use of the observer perspective in their retrieval of positive versus negative autobiographical memories. Memory.

[CR41] Neshat-Doost HT, Dalgleish T, Yule W, Kalantari M, Ahmadi SJ, Dyregrov A, Jobson L (2013). Enhancing autobiographical memory specificity through cognitive training: An intervention for depression translated from basic science. Clinical Psychological Science.

[CR42] Nolen-Hoeksema S, Morrow J (1991). A prospective study of depression and posttraumatic stress symptoms after a natural disaster: The Loma Prieta earthquake. Journal of Personality and Social Psychology.

[CR43] Nolen-Hoeksema S, Parker LE, Larson J (1994). Ruminative coping with depressed mood following loss. Journal of Personality and Social Psychology.

[CR44] Panahi Shahri, M. (1994). *Preliminary survey of validity, reliability and norm of state*-*trait anxiety inventory of Spielberger* (Persian). M.Sc. thesis in Psychology. Tehran: Tarbiat Modares University.

[CR45] Pearson DG, Deeprose C, Wallace-Hadrill SMA, Burnett Heyes S, Holmes EA (2013). Assessing mental imagery in clinical psychology: A review of imagery measures and a guiding framework. Clinical Psychological Review.

[CR46] Phillips WJ, Hine DW, Thorsteinsson EB (2010). Implicit cognition and depression: A meta-analysis. Clinical Psychology Review.

[CR47] Pictet A, Coughtrey AE, Mathews A, Holmes EA (2011). Fishing for happiness: The effects of positive imagery on interpretation bias and a behavioral task. Behaviour Research and Therapy.

[CR48] Rademaker, R. L., & Pearson, J. (2012). Training visual imagery: Improvements of metacognition, but not imagery strength. *Frontiers in Psychology,**3*, 224. 10.3389/fpsyg.2012.00224 PMC3392841 22787452

[CR49] Reisberg D, Pearson DG, Kosslyn SM (2003). Intuitions and introspections about imagery: The role of imagery experience in shaping an investigator’s theoretical views. Applied Cognitive Psychology.

[CR50] Rude SS, Wenzlaff RM, Gibbs B, Vane J, Whitney T (2002). Negative processing biases predict subsequent depressive symptoms. Cognition and Emotion.

[CR51] Salemink E, Hertel PT, Mackintosh B (2010). Interpretation training influences memory for prior interpretations. Emotion.

[CR52] Spielberger CD, Gorsuch RL, Lushene R, Vagg PR, Jacobs GA (1983). Manual for State-Trait Anxiety Inventory.

[CR101] Steer, R. A., & Beck, A. T. (2004). The Beck Depression Inventory-II. In W. E. Craighead & C. B. Nemeroff (Eds.), *The concise corsini encyclopedia of psychology and behavioral science* (3rd ed., pp. 104–105). New York: Wiley.

[CR53] Torkan H, Kalantari M, Neshat-Doost HT, Maroufi M, Talebi H (2012). Visual imagery and rumination as cognitive representations in major depressive disorder: A preliminary study. Interdisciplinary Journal of Contemporary Research in Business.

[CR54] Tran T, Hertel PT, Joormann J (2011). Cognitive bias modification: Induced interpretive biases affect memory. Emotion.

[CR55] Treynor W, Gonzalez R, Nolen-Hoeksema S (2003). Rumination reconsidered: A psychometric analysis. Cognitive Therapy and Research.

[CR56] Velten E (1968). A laboratory task of induction of mood states. Behaviour Research and Therapy.

[CR57] Watkins Ed R, Baeyens CB, Read R (2009). Concreteness training reduces dysphoria: Proof-of-principle for repeated cognitive bias modification in depression. Journal of Abnormal Psychology.

[CR58] Wenzlaff RM, Wegner DM, Pennebaker JW (1993). The mental control of depression: Psychological obstacles to emotional well-being. Handbook of mental control.

[CR59] Werner-Seidler A, Moulds ML (2012). Mood repair and processing mode in depression. Emotion.

[CR60] Williams AD, Blackwell SE, Mackenzie A, Holmes EA, Andrews G (2013). Combining imagination and reason in the treatment of depression: A randomized controlled trial of internet-based cognitive bias modification and internet-CBT for depression. Journal of Consulting and Clinical Psychology.

[CR61] Williams AD, Moulds ML (2007). Cognitive avoidance of intrusive memories: Recall vantage perspectives associations with depression. Behaviour Research and Therapy.

[CR62] Wittchen HU (2012). The burden of mood disorders. Science.

[CR63] World Health Organization (2008). The global burden of disease: 2004 update.

